# Myasthenia gravis: Diagnostic journey and therapeutic outcomes in patients followed at a Brazilian public tertiary center — A retrospective cohort study

**DOI:** 10.1371/journal.pone.0353883

**Published:** 2026-07-28

**Authors:** Diego Silva Figueiredo, Alberto Rolim Muro Martinez, Anamarli Nucci, Marcondes Cavalcante França Junior

**Affiliations:** Department of Neurology, University of Campinas, Campinas, São Paulo, Brazil; National Cerebral and Cardiovascular Center: Kokuritsu Junkankibyo Kenkyu Center, JAPAN

## Abstract

**Background:**

Myasthenia gravis (MG) is a heterogeneous autoimmune disorder of the neuromuscular junction. Diagnosis and management remain challenging in real-world settings, particularly in public health systems. Data from resource-constrained countries are limited. This study aimed to characterize the epidemiological, clinical, and therapeutic profile of MG patients followed in a Brazilian public tertiary center, emphasizing diagnostic delay and treatment outcomes.

**Methods:**

We conducted a retrospective single-center cohort study of 150 adult MG patients evaluated from 2024 to 2025. Clinical, immunological, therapeutic, and diagnostic-journey data were extracted from institutional electronic and paper medical records, and structured patient questionnaires, when available. Patients were classified as drug-responsive, corticosteroid-dependent, or drug-refractory using predefined operational criteria informed by international consensus guidance. Between-group comparisons used parametric or nonparametric tests, and categorical variables were analyzed with chi-square, Fisher’s exact, or Monte Carlo permutation chi-square tests, as appropriate.

**Results:**

The cohort had female predominance (65.3%), with a median age at onset 35.5 years (IQR 25–48). Among those tested, most were AChR antibody–positive (67.6%) and presented generalized MG (93.3%). Median diagnostic delay was 11 months, and 47.3% had delays ≥1 year. Refractory MG occurred in 14% and corticosteroid dependence in 10.6%. Documented myasthenic crisis and impending crisis occurred in 25.2% (35/139) and 27.3% (38/139), respectively; documented myasthenic crisis was more frequent in refractory than in drug-responsive patients (45.0% vs 21.0%). Treatment-related adverse events were documented in 47.5% (67/141); among affected patients, the most common were hyperglycemia (37.3%), osteoporosis/osteopenia (17.9%), and weight gain (16.4%).

**Conclusions:**

MG patients followed at this Brazilian public tertiary center showed clinical profiles broadly comparable to those reported in international cohorts, but diagnostic delays were frequent. The frequencies of refractory disease and corticosteroid dependence highlight the need for earlier recognition, specialized management, optimized steroid-sparing strategies, and improved access to comprehensive MG care.

## Introduction

Myasthenia gravis (MG) is an acquired autoimmune disorder of the neuromuscular junction in which antibodies impair acetylcholine-mediated transmission at the postsynaptic membrane. The majority of patients harbor antibodies against the acetylcholine receptor (AChR), whereas smaller subsets are positive for muscle-specific kinase (MuSK) or low-density lipoprotein receptor-related protein 4 (LRP4) antibodies. These immune-mediated mechanisms lead to variable impairment of neuromuscular transmission, producing fluctuating weakness. Clinically, MG encompasses a heterogeneous spectrum ranging from purely ocular involvement to generalized forms with bulbar, limb, and respiratory muscle weakness [1].

Epidemiological studies indicate a rising worldwide incidence and prevalence of MG. A recent systematic review and meta-analysis estimated a global prevalence of approximately 12.4 per 100,000 people (95% CI 10.6–14.5) and incidence figures ranging from 1.7 to 21.3 per million person-years [2,3]. However, contemporary population-based estimates vary substantially by country; for example, recent national prevalence estimates were 34.2 per 100,000 in France (2019), 39.3 per 100,000 in Germany (2019), and 37.0 per 100,000 in the United States (2021) [4–6]. Despite evolving epidemiologic data, substantial gaps remain in the diagnosis, therapeutic outcomes, and long-term disease control in real-world settings. Diagnostic delay is a critical concern: in a large European cohort of 387 patients, 27% experienced a delay of more than one year between symptom onset and MG diagnosis. Such delays have been associated with greater disease severity, worse health-related quality of life, and higher health-care resource use [7]. From a treatment perspective, while many patients achieve satisfactory control with first-line immunosuppressive therapy, a notable subset remains refractory or corticosteroid-dependent, with ongoing disease activity or treatment-related toxicity. The refractoriness rate in high-income countries has been reported at around 10–15% [8,9]. Comparable estimates from middle-income countries remain limited, constraining cross-setting comparisons**.** In addition, long-term corticosteroid exposure is associated with adverse events such as hyperglycemia, osteoporosis, weight gain, and impaired quality of life [10,11].

In Brazil, where access to neurologic subspecialists, advanced diagnostic testing, and novel therapies may be constrained within the public health system, robust data on the clinical course, diagnostic journey, and therapeutic outcomes of MG remain scarce. To address these gaps, we conducted a retrospective cohort study of MG patients managed in a Brazilian public tertiary center. The objectives were to (1) delineate the epidemiological, immunological, and clinical characteristics of the cohort; (2) characterize the diagnostic journey—including time from symptom onset to diagnosis, number of physicians consulted, and occurrence of misdiagnoses—and contextualize these findings alongside published international data; and (3) evaluate therapeutic outcomes, including rates of refractoriness, corticosteroid dependence, and treatment-related adverse events. By doing so, we aimed to provide insights into MG management in a middle-income country context and identify opportunities to optimize diagnosis and care pathways.

## Methods

### Study design and patients

This retrospective cohort study was conducted at the Neuromuscular Diseases Outpatient Clinic of the State University of Campinas (UNICAMP, Brazil), a public tertiary referral center. Consecutive adult patients (≥18 years) with a confirmed diagnosis of MG, irrespective of the year of diagnosis, were included if they attended follow-up visits between July 2024 and September 2025. A total of 150 patients met the inclusion criteria. Data collection took place between September 30, 2024, and September 30, 2025.

The diagnosis of MG was established based on characteristic clinical features in combination with at least one supportive criterion: positivity for anti-acetylcholine receptor (AChR) or anti-MuSK antibodies, abnormal electrophysiological findings on repetitive nerve stimulation or single-fiber electromyography, or unequivocal pharmacological response to acetylcholinesterase inhibitors. Patients with alternative neuromuscular diagnoses were excluded. Individuals with incomplete records were retained to minimize selection bias; variables with insufficient information were coded as missing and analyzed using available-case denominators.

### Variables and definitions

Data were extracted from the institutional electronic and paper medical records and from structured patient questionnaires. Collected variables included demographic data (sex, race/color, and age at symptom onset) and diagnostic journey metrics (time from symptom onset to diagnosis in months, number of physicians consulted before diagnosis, and reported prior misdiagnoses). Age at symptom onset, diagnostic delay, number of physicians consulted, and misdiagnosis were primarily patient-reported through the structured questionnaire; when more precise information was available in the medical records, chart-based data were preferentially used. Diagnostic delay was defined as the reported interval, in months, between the first MG-related symptoms and clinical or laboratory confirmation of MG diagnosis. Disease duration was calculated as the interval between symptom onset and the final date of data collection. The number of physicians consulted before diagnosis was reported in predefined categories. Misdiagnosis was defined as an alternative diagnosis reportedly communicated by a physician before MG confirmation.

Clinical variables comprised the Myasthenia Gravis Foundation of America (MGFA) clinical classification and MGFA Postintervention Status (PIS) at the time of the last evaluation, disease phenotype (ocular, bulbar, or generalized), antibody status (AChR, MuSK, or seronegative), thymic pathology (hyperplasia or thymoma), thymectomy, and presence of major comorbidities. Electrophysiological findings from nerve conduction studies, repetitive nerve stimulation, and single-fiber electromyography were obtained from prior diagnostic reports; all studies were performed in accordance with the standards of the International Federation of Clinical Neurophysiology (IFCN).

Outcome-related variables included the occurrence of at least one hospitalization, documented myasthenic crisis, documented impending crisis, and any adverse event related to immunosuppressive or corticosteroid therapy, as documented in medical records. Myasthenic crisis was defined as respiratory failure attributable to MG requiring ventilatory support. Impending crisis was defined as documented clinically significant bulbar and/or respiratory worsening judged by the treating team to represent imminent risk of respiratory failure and requiring hospital admission, urgent hospital-based intervention, and/or rescue therapy, without established respiratory failure requiring ventilatory support. Myasthenic crisis and impending crisis were analyzed separately.

### Treatment groups

Patients were categorized into three groups according to treatment response and corticosteroid dependence, based on longitudinal chart review and treatment status at the last clinical evaluation:

1. Drug-responsive (DR) — patients who did not meet criteria for refractory MG and who were managed with no more than one steroid-sparing agent and a maintenance prednisone-equivalent dose <20 mg/day.
2. Corticosteroid-dependent (C) — patients who did not meet criteria for refractory MG but had been receiving corticosteroid therapy for ≥6 months for symptom control and were unable to taper and maintain a prednisone-equivalent dose <20 mg/day with or without concomitant immunosuppressive therapy.
3. Drug-refractory (R) — patients who met the operational definition of refractory disease, as detailed below.

#### Definition of refractory disease.

Refractoriness was defined according to the 2021 International Consensus Guidance for Management of Myasthenia Gravis. Patients were considered refractory when they had persistent disabling symptoms, clinical worsening, or lack of meaningful clinical improvement despite adequate treatment with corticosteroids and at least two distinct non-steroidal immunosuppressive agents administered at therapeutic doses and for a clinically appropriate duration, according to chart documentation and treating-physician assessment. Treatment-limiting adverse events or intolerance to conventional therapy were also accepted as criteria for refractoriness when they prevented continued use, dose optimization, or escalation of otherwise appropriate immunosuppressive treatment, as jointly determined by the patient and treating physician [12]. Retrospectively assessed MGFA Postintervention Status (PIS), particularly unchanged, worsened, or exacerbation status, was used as a supportive criterion for classification. This operational definition was used to classify patients in the drug-refractory (R) group for all subsequent analyses. The individual criteria supporting refractory classification are detailed in S7 Table.

### Statistical analysis

Statistical analyses were performed using Python (version 3.11) with the pandas, SciPy, and statsmodels libraries. Continuous variables were expressed as mean ± standard deviation (SD) or median and interquartile range (IQR), according to distribution. Normality was assessed with the Shapiro–Wilk test, and equality of variances with Levene’s test. Between-group comparisons were performed using the Student’s t test or Welch’s t test for normally distributed variables, and the Mann–Whitney U test for non-parametric data.

Categorical variables were reported as absolute and relative frequencies and compared using the chi-square test or Fisher’s exact test, as appropriate. When expected frequencies were <5 in more than 20% of cells, p values were estimated by a Monte Carlo resampling procedure with 20,000 permutations. For 2 × 2 contingency tables, odds ratios (OR) and 95% confidence intervals (95% CI) were calculated, applying the Haldane–Anscombe correction when zero-cell counts occurred. For categorical variables with more than two levels, categories with fewer than five observations were collapsed into an “Other” category before analysis.

Three clinical groups (R, C, and DR) were described in all summary tables, and inferential analyses focused on the pairwise comparisons R vs DR and C vs DR, with corresponding p values reported. These comparisons were considered exploratory. In an additional exploratory analysis, binary logistic regression was used to investigate factors associated with a diagnostic delay of 12 months or longer. The multivariable model included age at symptom onset, sex, and race/color, selected a priori based on clinical relevance and data availability. Age at symptom onset was modeled per 10-year increase, sex as female versus male, and race/color as White versus non-White.

Separate exploratory binary logistic regression models were fitted for hospitalization, documented myasthenic crisis, impending myasthenic crisis, and treatment-related adverse events, with adjustment for disease duration. Models were fitted separately for the R vs DR and C vs DR comparisons and included treatment-response group and disease duration in years as independent variables. Adjusted odds ratios (aOR), 95% confidence intervals, and p values from the diagnostic-delay and cumulative-outcome models were reported. Complete results of the disease-duration-adjusted outcome models are presented in S8 Table.

Variables with missing data were analyzed using available-case denominators, without imputation. Denominators for major variables were summarized in S5 Table. To assess missingness patterns, patients with complete and incomplete data for diagnostic-journey and cumulative outcome variables were compared in supplementary analyses summarized in S6 Table. All tests were two-tailed, and statistical significance was defined as p < 0.05.

### Ethical standards

The study was approved by the Research Ethics Committee of the State University of Campinas (UNICAMP) (approval no. 7.106.694; CAAE: 81196324.6.0000.5404) and conducted in accordance with the ethical standards of the 1964 Declaration of Helsinki and its later amendments. Written informed consent was obtained from all participating patients whenever possible. For patients who could not be directly contacted, data use was authorized under a Term of Consent for Use of Data (TCUD) approved by the Ethics Committee. Patient confidentiality was maintained by anonymizing all records prior to data analysis.

## Results

### Cohort profile

Between July 2024 and September 2025, a total of 150 patients with myasthenia gravis (MG) were included. The cohort comprised 98 (65.3%) females and 52 (34.7%) males, with a median age at disease onset of 35.5 years (interquartile range [IQR] 25–48). Most patients self-identified as White (74.7%), followed by Brown (15.3%), Black (9.3%), and Asian (0.7%). The predominant clinical phenotype was generalized MG (93.3%), followed by ocular (5.3%) and bulbar (1.3%) forms. Regarding immunological status, 67.6% (73/108) were AChR antibody–positive, 6.5% (7/108) MuSK antibody–positive, and 21.3% (23/108) seronegative. Thymic pathology was documented in 24.6% of patients with available thymic assessment (34/138), including thymoma in 13.8% (19/138) and thymic hyperplasia in 10.9% (15/138). A total of 36.0% (54/150) of patients underwent thymectomy, performed either for thymic pathology or as part of disease management, irrespective of histological findings. During disease course, documented myasthenic crisis and documented impending crisis occurred in 25.2% (35/139) and 27.3% (38/139) of patients, respectively. Coexisting autoimmune disorders were present in 8.0% of patients, most frequently autoimmune hypothyroidism (6.7%), systemic lupus erythematosus (0.7%), and rheumatoid arthritis (0.7%). Table 1 summarizes baseline demographic and clinical characteristics.

**Table 1:** Baseline demographic and clinical characteristics of the cohort (n = 150).

Characteristic	Category	Total
**Sex**	Female	98 (65.3)
	Male	52 (34.7)
**Race/color**	White	112 (74.7)
	Brown	23 (15.3)
	Black	14 (9.3)
	Asian	1 (0.7)
**Age at symptom onset, years, median (IQR)**	Overall	35.5 (25.0–48.0)
	Female	31.0 (24.0–43.5)
	Male	44.0 (30.8–59.3)
**Phenotype**	Generalized	140 (93.3)
	Ocular	8 (5.3)
	Bulbar	2 (1.3)
**MGFA clinical classification**	I	35 (23.3)
	II	70 (46.7)
	III	13 (8.7)
	IV	0
	V	0
	Not applicable — no active manifestations	32 (21.3)
**MGFA-PIS classification, N = 146**	Improved	90 (61.6)
	Unchanged	14 (9.6)
	Worsened	5 (3.4)
	Exacerbation	3 (2.1)
	CSR	7 (4.8)
	PR	17 (11.6)
	MM	10 (6.8)
**Immunological status, N = 108**	AChR-Ab+	73 (67.6)
	MuSK-Ab+	7 (6.5)
	Double-positive	1 (0.9)
	Seronegative	23 (21.3)
	Double-negative	4 (3.7)
**Other autoimmune disease**	Any	12 (8.0)
	Autoimmune hypothyroidism	10 (6.7)
	Systemic lupus erythematosus	1 (0.7)
	Rheumatoid arthritis	1 (0.7)
**Thymic pathology, N = 138**	Thymoma	19 (13.8)
	Thymic hyperplasia	15 (10.9)
**Thymectomy**		54 (36.0)
**≥1 hospitalization, N = 113**		47 (41.6)
**Documented myasthenic crisis/impending crisis, N = 139**	Myasthenic crisis	35 (25.2)
	Impending crisis	38 (27.3)

Data are expressed as median (IQR) or n (%). Denominators are shown when data were not available for the full cohort. AChR-Ab + : acetylcholine receptor antibody-positive; MuSK-Ab + : muscle-specific kinase antibody-positive; DN: double seronegative (AChR − /MuSK−); SN: seronegative (AChR− with MuSK not tested/unavailable); DP: double seropositive (AChR + /MuSK+).

### Diagnostic journey

**Fig 1:**
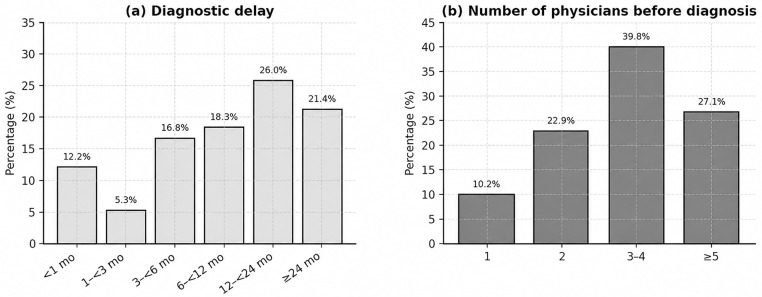
Diagnostic journey in patients with myasthenia gravis. (A) Distribution of diagnostic delay intervals (n = 131). (B) Number of physicians consulted before diagnosis (n = 118). Values represent the percentage of patients within each category. Diagnostic delay was defined as the time from symptom onset to confirmed diagnosis.

**Fig 2:**
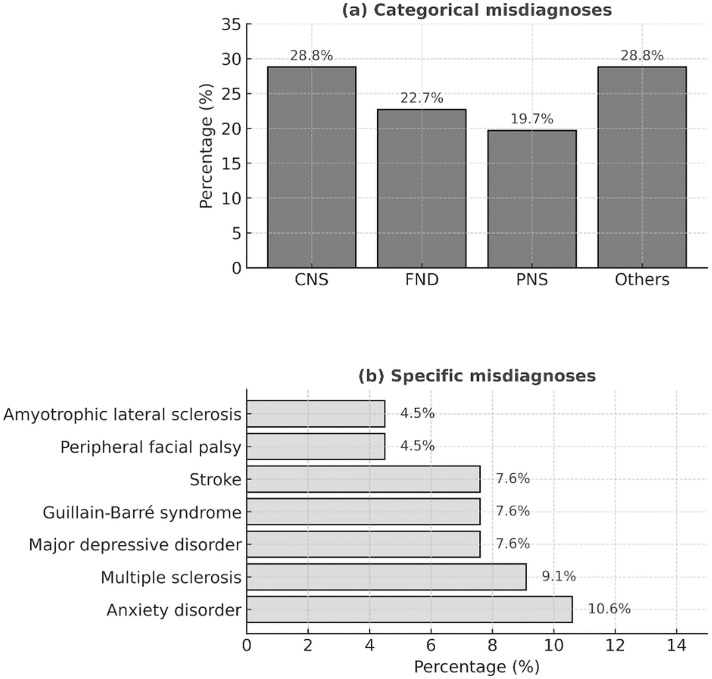
Categorical and specific misdiagnoses in patients with myasthenia gravis. (A) Distribution of categorical misdiagnoses, including central nervous system (CNS), functional neurological disorder (FND), peripheral nervous system (PNS), and other disorders. (B) Most frequent specific misdiagnoses reported, including anxiety disorder, multiple sclerosis, major depressive disorder, and Guillain-Barré syndrome. Values represent the percentage of all reported misdiagnoses (n = 66). One patient reported more than one misdiagnosis.

The median time from symptom onset to confirmed diagnosis was 11 months (IQR 4–18). Nearly half of the cohort (47.3%; 62/131) experienced a diagnostic delay of 12 months or longer, while only 34.3% were diagnosed within six months (Table 2 and Fig 1A). In an exploratory multivariable logistic regression, older age at symptom onset was associated with lower odds of a diagnostic delay of 12 months or longer (adjusted OR per 10-year increase, 0.79, 95% CI 0.63–0.99; p = 0.037). No significant associations were observed for female sex (adjusted OR 1.14, 95% CI 0.52–2.49; p = 0.747) or White versus non-White race/color (adjusted OR 0.95, 95% CI 0.42–2.11; p = 0.891). The number of physicians consulted before diagnosis varied considerably: most patients (39.8%) received their diagnosis only after consulting three to four physicians, whereas 10.2% were correctly diagnosed at the first visit (Fig 1B). Overall, 55.6% of patients reported at least one previous misdiagnosis. The most frequent misdiagnosis categories were central nervous system disorders (28.8%) and functional neurological disorders (22.7%). The specific conditions most often reported were anxiety disorder (10.6%), multiple sclerosis (9.1%), and major depressive disorder (7.6%) (Figs 2A and 2B and S4 Table). A myasthenic crisis or impending crisis was the initial presentation in 25.7% of patients.

**Table 2:** Diagnostic journey of patients with myasthenia gravis.

Diagnostic delay, n = 131	n (%)
< 1 mo	16 (12.2)
1– < 3 mo	7 (5.3)
3– < 6 mo	22 (16.8)
6– < 12 mo	24 (18.3)
12– < 24 mo	34 (26.0)
≥ 24 mo	28 (21.4)
**Number of physicians, n = 118**	
1	12 (10.2)
2	27 (22.9)
3–4	47 (39.8)
≥ 5	32 (27.1)
**Misdiagnosis, n = 117**	65 (55.6)
**Onset with myasthenic crisis or impending crisis, n = 101**	26 (25.7)

Distribution of diagnostic delay and number of physicians consulted before diagnosis, as well as the proportions of misdiagnosis and myasthenic crisis or impending crisis at disease onset. Data are presented as No. (%). Sample sizes (n) are shown for each item and may vary due to missing data. Abbreviations: mo, months.

### Therapeutic outcomes

**Fig 3:**
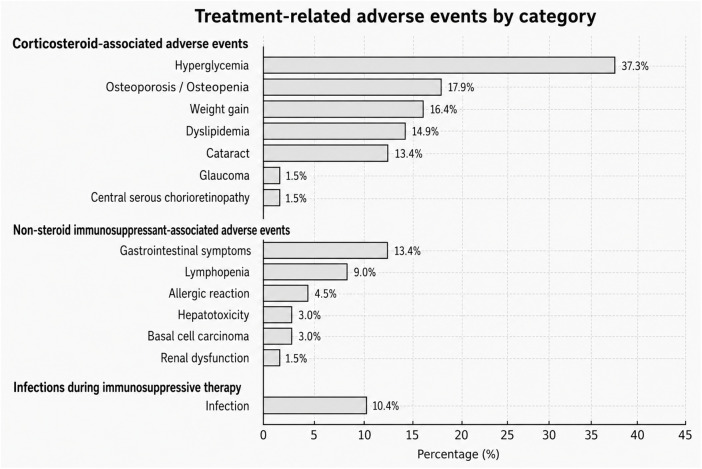
Treatment-related adverse events by category. Specific adverse events are grouped as corticosteroid-associated adverse events, non-steroid immunosuppressant-associated adverse events, and infections during immunosuppressive therapy. Values represent the percentage of patients with at least one documented treatment-related adverse event (n = 67). More than one adverse event could be documented per patient. The categories reflect clinical associations identified through retrospective chart review and do not imply definite causality. Detailed counts are provided in S3 Table.

At last follow-up, 75.3% of patients were classified as drug-responsive, 14.0% as refractory, and 10.6% as corticosteroid-dependent. Although not statistically significant, MuSK antibody positivity was numerically more frequent among refractory patients (11.1%) compared with drug-responsive (5.3%) and corticosteroid-dependent (6.7%) groups. Patients in the refractory group more often experienced diagnostic delays of 24 months or longer compared with drug-responsive individuals (57.9% vs 14.1%; OR 8.35, 95% CI 2.86–24.39; p < 0.001). Overall, 47.5% (67/141) of patients had at least one documented treatment-related adverse event. Adverse events occurred in 39.6% of drug-responsive patients, 70.0% of refractory patients, and 73.3% of corticosteroid-dependent patients. Compared with drug-responsive patients, the odds of treatment-related adverse events were higher in both the refractory group (OR 3.56, 95% CI 1.27–9.98; p = 0.015) and the corticosteroid-dependent group (OR 4.19, 95% CI 1.25–14.04; p = 0.024). Among patients with ≥1 adverse event (n = 67), the most commonly documented events were hyperglycemia (37.3%), osteoporosis/osteopenia (17.9%), and weight gain (16.4%) (Fig 3 and S3 Table). At least one hospitalization occurred in 66.7% of refractory patients, compared with 39.1% of drug-responsive and 27.3% of corticosteroid-dependent patients. Refractory patients had higher estimated odds of hospitalization than drug-responsive patients, although the confidence interval included the null value (OR 3.12, 95% CI 0.98–9.91; p = 0.054), whereas no significant difference was observed between corticosteroid-dependent and drug-responsive patients (p = 0.527). Documented myasthenic crisis occurred more frequently in refractory than in drug-responsive patients (45.0% vs 21.0%; OR 3.09, 95% CI 1.14–8.38; p = 0.044), whereas no significant difference was observed between corticosteroid-dependent and drug-responsive patients (28.6% vs 21.0%; p = 0.503). Documented impending crisis did not differ significantly between refractory and drug-responsive patients (25.0% vs 26.7%; p = 1.000) or between corticosteroid-dependent and drug-responsive patients (35.7% vs 26.7%; p = 0.529). Median disease duration was 11.5 years (IQR 4.4–21.5) in drug-responsive patients, 15.8 years (IQR 10.1–22.9) in refractory patients, and 8.5 years (IQR 3.8–14.6) in corticosteroid-dependent patients, with no statistically significant differences in pairwise comparisons (R vs DR: p = 0.114; C vs DR: p = 0.389). In exploratory logistic regression models adjusted for disease duration, documented myasthenic crisis remained more frequent among refractory than drug-responsive patients (adjusted OR 3.32, 95% CI 1.20–9.21; p = 0.021). Treatment-related adverse events also remained more frequent among refractory (adjusted OR 3.27, 95% CI 1.14–9.31; p = 0.027) and corticosteroid-dependent patients (adjusted OR 4.76, 95% CI 1.39–16.28; p = 0.013) than among drug-responsive patients. Differences in hospitalization and impending crisis remained non-significant after adjustment (S8 Table). No statistically significant differences were observed among groups in sex distribution, age at onset, race, immunological status, thymic pathology, or presence of comorbid autoimmune diseases (p > 0.05 for all comparisons). Odds ratios with 95% confidence intervals and p values are provided in S1 Table. Prior immunosuppressive treatments stratified by therapeutic response group are shown in S2 Table. The clinical, immunological, and outcome profile by treatment-response group is summarized in Table 3.

**Table 3:** Clinical, immunological, and outcome profile of drug-responsive, drug-refractory, and corticosteroid-dependent myasthenia gravis.

	Drug-responsive n = 113	Drug-refractory n = 21	Corticosteroid- dependentn = 16
**Sex**			
Female	75 (66.4)	14 (66.7)	9 (56.2)
Male	38 (33.6)	7 (33.3)	7 (43.8)
Age at symptom onset, years, median (IQR)	36.0 (27.0-47.0)	33.0 (20.0-48.0)	35.5 (23.2-60.5)
**Race/color**			
White	85 (75.2)	14 (66.7)	13 (81.2)
Black	10 (8.8)	3 (14.3)	1 (6.2)
Brown	17 (15.0)	4 (19.0)	2 (12.5)
Asian	1 (0.9)	0 (0.0)	0 (0.0)
**Phenotype**			
Ocular	8 (7.1)	0 (0.0)	0 (0.0)
Generalized	104 (92.0)	20 (95.2)	16 (100.0)
Bulbar	1 (0.9)	1 (4.8)	0 (0.0)
**MGFA clinical class at last evaluation**			
I	30 (26.5)	2 (9.5)	3 (18.8)
II	47 (41.6)	14 (66.7)	9 (56.2)
III	6 (5.3)	5 (23.8)	2 (12.5)
IV	0 (0.0)	0 (0.0)	0 (0.0)
V	0 (0.0)	0 (0.0)	0 (0.0)
Not applicable — no active manifestations	30 (26.5)	0 (0.0)	2 (12.5)
**MGFA-PIS at last evaluation**	n = 110	n = 21	n = 15
CSR	7 (6.4)	0 (0.0)	0 (0.0)
PR	17 (15.5)	0 (0.0)	0 (0.0)
MM	8 (7.3)	0 (0.0)	2 (13.3)
Improved	71 (64.5)	6 (28.6)	13 (86.7)
Worsened	2 (1.8)	3 (14.3)	0 (0.0)
Exacerbation	2 (1.8)	1 (4.8)	0 (0.0)
Unchanged	3 (2.7)	11 (52.4)	0 (0.0)
**Immunological status**	n = 75	n = 18	n = 15
AChR-Ab+	50 (66.7)	13 (72.2)	10 (66.7)
MuSK-Ab+	4 (5.3)	2 (11.1)	1 (6.7)
DP	0	1 (5.6)	0
DN	3 (4.0)	0	1 (6.7)
SN	18 (24.0)	2 (11.1)	3 (20.0)
**Other autoimmune disease**			
≥ 1 other autoimmune disease	9 (8.0)	3 (14.3)	0 (0.0)
**Thymic pathology**	n = 103	n = 19	n = 16
Thymoma	15 (14.6)	2 (10.5)	2 (12.5)
Hyperplasia	12 (11.7)	2 (10.5)	1 (6.2)
Thymectomy	40 (35.4)	10 (47.6)	4 (25.0)
**Hospitalization**			
≥ 1 hospitalization	n = 87 34 (39.1)	n = 15 10 (66.7)	n = 11 3 (27.3)
**Documented myasthenic crisis/impending crisis**	n = 105	n = 20	n = 14
Myasthenic crisis	22 (21.0)	9 (45.0)	4 (28.6)
Impending crisis	28 (26.7)	5 (25.0)	5 (35.7)
**Adverse events**	n = 106	n = 20	n = 15
≥ 1 adverse event	42 (39.6)	14 (70.0)	11 (73.3)
**Disease duration**			
Years, median (IQR)	11.5 (4.4-21.5)	15.8 (10.1-22.9)	8.5 (3.8-14.6)

Baseline characteristics, immunological status, thymic pathology/interventions, and outcome-related variables are presented by treatment-response group. Data are shown as No. (%) unless otherwise indicated; age at onset and disease duration are presented as median (IQR). Sample sizes (n) are provided for each variable and may vary due to missing data.

† Immunological status refers to antibody profile: AChR-Ab + , acetylcholine receptor antibody–positive; MuSK-Ab + , muscle-specific kinase antibody–positive; DP, double seropositive (AChR + /MuSK+); DN, double seronegative (AChR − /MuSK−); SN, seronegative (AChR − /MuSK unknown).

‡ MGFA, Myasthenia Gravis Foundation of America clinical classification.

§ MGFA-PIS, MGFA Postintervention Status; CSR, complete stable remission; PR, pharmacologic remission; MM, minimal manifestations.

### Missing data analysis

In supplementary missing-data analyses, patients with incomplete diagnostic-journey data had a longer median disease duration than those with complete data (16.9 vs 9.8 years; p = 0.002). Patients with incomplete cumulative outcome data also had a longer median disease duration than those with complete data (15.4 vs 9.9 years; p = 0.007). No statistically significant differences were observed in age at symptom onset, sex, race/color, phenotype, documented AChR or MuSK positivity, or treatment-group distribution between patients with complete and incomplete data (S6 Table).

## Discussion

This study provides a comprehensive characterization of patients with myasthenia gravis (MG) followed in a Brazilian public tertiary center, emphasizing epidemiological features, diagnostic trajectory, and treatment outcomes. The clinical and immunological distribution of our cohort was consistent with that reported in international cohorts, with female predominance and predominant anti-AChR antibody positivity. These similarities suggest that, despite structural differences across health systems, the clinical and immunological profile observed in this Brazilian tertiary-center cohort resembles that reported in North American and European populations.

A central finding concerns the diagnostic journey. The median delay between symptom onset and confirmed diagnosis was 11 months, and 47.3% of patients experienced delays of 12 months or longer—including 26.0% with delays of 12 to <24 months and 21.4% with delays of ≥24 months. These figures are considerable when contextualized alongside recent international data. In a large European cohort, 27% of patients experienced diagnostic delays greater than one year [7], while the mean delay in a Danish tertiary center was approximately 331 days (~11 months) [13]. Conversely, an Australian multicenter study reported a median delay of 102 days (~3.4 months) [14]. Together, these findings illustrate variation in diagnostic timeliness across healthcare settings, although direct comparisons are limited by differences in cohort composition and study methodology. In the Brazilian public context, potential contributors may include limited access to neurologists, restricted availability of electrophysiological and antibody testing, and low disease awareness among primary care physicians, although these factors were not directly assessed in our study. Addressing such barriers may help shorten the diagnostic journey and reduce morbidity associated with delayed treatment.

Within the Brazilian public health system, therapeutic management of myasthenia gravis remains largely limited to conventional immunosuppressive agents—mainly corticosteroids, azathioprine, cyclosporine, and, in refractory cases, cyclophosphamide, as outlined in the national clinical protocol for MG [15]. Although some advanced or targeted therapies (e.g., complement inhibitors, FcRn antagonists, and B-cell–depleting agents) have regulatory approval in Brazil, they are not included in the current national MG protocol for routine use within the public health system (SUS), and access in public-sector care remains limited. Intravenous immunoglobulin (IVIG) and plasma exchange are generally used as short-term therapies for myasthenic crisis, severe exacerbations, or other situations requiring a rapid clinical response, rather than for long-term maintenance. This restricted therapeutic landscape may contribute to corticosteroid dependence and emergence of treatment-related adverse events.

Regarding therapeutic outcomes, 14% of patients fulfilled criteria for refractory MG, a prevalence consistent with international estimates of 10–15% [8,9]. Refractory patients more frequently experienced documented myasthenic crisis and treatment-related adverse events than drug-responsive patients. Hospitalization was also numerically more frequent among refractory patients, although the pairwise comparison did not reach statistical significance. These associations may in part reflect greater disease severity and treatment complexity. Although cumulative outcomes are exposure-time dependent, disease duration did not differ significantly between treatment groups. Anti-MuSK positivity was numerically more frequent among refractory patients; however, this difference did not reach statistical significance and should be interpreted cautiously given the small subgroup sizes. Previous studies have associated MuSK-positive MG with greater disease severity and a less favorable response to conventional immunosuppression [16], but no firm conclusions regarding this association can be drawn from our findings.

The corticosteroid-dependent subgroup represented a distinct clinical profile from drug-responsive and refractory MG, comprising patients who did not meet criteria for refractoriness but had received corticosteroid therapy for at least six months and were unable to taper and maintain prednisone-equivalent doses below 20 mg/day. Treatment-related adverse events were frequent in this subgroup; across patients with documented adverse events, the most common were hyperglycemia, osteoporosis/osteopenia, and weight gain, reflecting the metabolic and musculoskeletal toxicity of chronic corticosteroid use. These complications may increase morbidity and impair quality of life. The relatively low proportion of patients achieving pharmacological or complete remission underscores the persistent challenges in achieving sustained disease control. This therapeutic gap underscores the need for effective steroid-sparing strategies and broader access to specialized MG care, particularly for patients with refractory or corticosteroid-dependent disease.

Despite the limitations inherent to its retrospective and single-center design, this study provides one of the few systematic analyses of MG patients followed at a Brazilian public tertiary center. Diagnostic-journey data were partly based on patient reports and may therefore be subject to recall and information bias. Incomplete documentation resulted in variable denominators for some outcomes. Patients with incomplete diagnostic-journey or cumulative-outcome data had longer disease duration, which may reflect greater documentation gaps in patients with longer disease courses. The tertiary referral setting and the inclusion of patients attending follow-up may also have favored individuals with more severe or complex disease while underrepresenting those with mild disease, those lost to follow-up, or those who died. In addition, the high proportion of White participants relative to national and local demographics may reflect referral patterns, differential access to healthcare, socioeconomic factors, or characteristics of the center’s catchment area. The modest sample size, particularly in the refractory and corticosteroid-dependent subgroups, further limited statistical power, and between-group comparisons should therefore be considered exploratory.

The consecutive inclusion of patients and the use of standardized analytical procedures improved the consistency of data collection and analysis. Future multicenter and longitudinal studies involving more diverse populations are warranted to confirm these findings, investigate prognostic factors associated with refractoriness, and assess long-term treatment outcomes and safety.

## Conclusion

The clinical and immunological profile of patients followed at this Brazilian public tertiary center was broadly consistent with international reports, but diagnostic delay remained substantial, with nearly half of patients diagnosed at least one year after symptom onset. Refractory patients experienced a higher frequency of documented myasthenic crisis, while hospitalization was numerically more frequent among these patients. Treatment-related adverse events were particularly frequent in both refractory and corticosteroid-dependent patients. These findings underscore the need for earlier recognition, broader access to specialized MG care, and effective steroid-sparing strategies to improve disease control and reduce treatment-related morbidity.

## Supporting information

S1 Table Statistical comparisons between treatment-response groups. Comparisons were performed using Fisher’s exact test for binary variables, chi-square test or Monte Carlo permutation chi-square test for categorical variables with more than two levels, and the Mann–Whitney U test for continuous variables. Odds ratios (OR) with 95% confidence intervals (CI) were calculated for 2 × 2 contingency tables, applying the Haldane–Anscombe correction when zero-cell counts were present. All p values are two-tailed; significance threshold was set at p < 0.05.
(DOCX)

S2 Table Prior immunosuppressive treatment by treatment-response group. Values indicate the number and proportion of patients within each therapeutic-response group who had previously received the listed immunosuppressive agent before the last clinical evaluation.
(DOCX)

S3 Table Specific treatment-related adverse events. Values represent the percentage of patients among those with at least one documented treatment-related adverse event (n = 67). More than one adverse event could be documented per patient. Events were grouped according to treatment exposure and known toxicity profiles as corticosteroid-associated adverse events, non-steroid immunosuppressant-associated adverse events, or infections during immunosuppressive therapy. These categories indicate clinical association in a retrospective chart review and do not imply definite causality.
(DOCX)

S4 Table Specific misdiagnoses in patients with myasthenia gravis. Values represent the percentage of all reported misdiagnoses (n = 66). One patient reported more than one misdiagnosis. COPD, chronic obstructive pulmonary disease; GERD, gastroesophageal reflux disease.
(DOCX)

S5 Table Data availability and denominators for major variables, overall and by treatment group. Values indicate the number of patients with available or classifiable information for each variable. Percentages represent data availability, not the frequency of the corresponding clinical finding.
(DOCX)

S6 Table Comparison of patients with complete and incomplete data for missingness pattern assessment. Complete diagnostic-journey data were defined as availability of diagnostic delay, number of physicians consulted before diagnosis, and previous misdiagnosis. Complete cumulative outcome data were defined as availability of lifetime myasthenic crisis history, hospitalization history, and treatment-related adverse events.
(DOCX)

S7 Table Individual criteria supporting classification of drug-refractory myasthenia gravis. The table details adequate corticosteroid exposure, adequately tested nonsteroidal immunosuppressive agents, clinical response, treatment-limiting adverse events or intolerance, and the final basis for refractory classification for each patient.
(DOCX)

S8 Table Exploratory logistic regression analyses of cumulative therapeutic outcomes adjusted for disease duration. Separate binary logistic regression models were fitted for each outcome and pairwise treatment-group comparison. Models included treatment-response group and disease duration in years. Adjusted odds ratios refer to the treatment-group comparison shown, with drug-responsive patients as the reference group.
(DOCX)
