# Supplementary material for: Myasthenia gravis: Diagnostic journey and therapeutic outcomes in patients followed at a Brazilian public tertiary center — A retrospective cohort study
Source: PLoS One. 2026 Jul 28;21(7):e0353883. doi: 10.1371/journal.pone.0353883 (PMC13411926; doi:10.1371/journal.pone.0353883)
Supplement: S1 Table — Comparisons were performed using Fisher’s exact test for binary variables, chi-square test or Monte Carlo permutation chi-square test for categorical variables with more than two levels, and the Mann–Whitney U test for continuous variables. Odds ratios (OR) with 95% confidence intervals (CI) were calculated for 2 × 2 contingency tables, applying the Haldane–Anscombe correction when zero-cell counts were present. All p values are two-tailed; significance threshold was set at p < 0.05. (DOCX) [file pone.0353883.s001.docx]

**S1 Table. Statistical comparisons between treatment-response groups**

| **Variable** | **p (R vs DR)** | **OR (95% CI) R vs DR** | **p (C vs DR)** | **OR (95% CI) C vs DR** | **p (R vs DR+C)** | **OR (95% CI) R vs DR+C** |
| --- | --- | --- | --- | --- | --- | --- |
| Age at symptom onset | 0.713 | — | 0.943 | — | 0.690 | — |
| Disease duration, years | 0.114 | — | 0.389 | — | 0.081 | — |
| Sex | 1.000 | — | 0.418 | — | 1.000 | — |
| Race/color | 0.677 | — | 1.000 | — | 0.658 | — |
| Time-to-diagnosis | 0.001 | — | 0.252 | — | 0.002 | — |
| Diagnostic delay >24 months | <0.001 | 8.35 (2.86–24.39) | 0.414 | 1.82 (0.45–7.45) | <0.001 | 7.68 (2.70–21.89) |
| Phenotype | 0.200 | — | 0.641 | — | 0.170 | — |
| MGFA classification | <0.001 | — | 0.341 | — | 0.001 | — |
| AChR-Ab+ | 0.782 | 1.30 (0.42–4.06) | 1.000 | 1.00 (0.31–3.24) | 0.786 | 1.30 (0.42–3.99) |
| MuSK-Ab+ | 0.328 | 2.22 (0.37–13.18) | 1.000 | 1.27 (0.13–12.21) | 0.330 | 2.12 (0.38–11.92) |
| Thymoma | 1.000 | 0.69 (0.14–3.30) | 1.000 | 0.84 (0.17–4.07) | 1.000 | 0.71 (0.15–3.33) |
| Other autoimmune disease | 0.401 | 1.93 (0.48–7.80) | 0.600 | 0.36 (0.02–6.54) | 0.376 | 2.22 (0.55–8.99) |
| ≥1 Adverse events | 0.015 | 3.56 (1.27–9.98) | 0.024 | 4.19 (1.25–14.04) | 0.051 | 2.99 (1.08–8.32) |
| ≥1 Hospitalization | 0.054 | 3.12 (0.98–9.91) | 0.527 | 0.58 (0.14–2.36) | 0.048 | 3.30 (1.05–10.40) |
| ≥1 Myasthenic crisis | 0.044 | 3.09 (1.14–8.38) | 0.503 | 1.51 (0.43–5.27) | 0.048 | 2.93 (1.10–7.82) |
| ≥1 Impending crisis | 1.000 | 0.92 (0.30–2.76) | 0.529 | 1.53 (0.47–4.95) | 1.000 | 0.87 (0.29–2.58) |
| Myasthenic crisis or impending crisis at onset | 0.270 | 2.14 (0.55–8.39) | 0.723 | 1.20 (0.29–5.00) | 0.276 | 2.09 (0.54–8.09) |

Comparisons were performed using Fisher’s exact test for binary variables, chi-square test or Monte Carlo permutation chi-square test for categorical variables with more than two levels, and the Mann–Whitney U test for continuous variables. For categorical variables with more than two levels, categories with fewer than five observations were collapsed into an “Other” category before analysis; when expected frequencies were <5 in more than 20% of cells, p-values were estimated using a Monte Carlo resampling procedure with 20,000 permutations. Odds ratios (OR) with 95% confidence intervals (CI) were calculated for 2×2 contingency tables, applying the Haldane–Anscombe correction when zero-cell counts were present. DR+C denotes the combined non-refractory group. ORs are expressed for the first-named group relative to the second. All p values are two-tailed; the significance threshold was p < 0.05.
