# Supplementary material for: Myasthenia gravis: Diagnostic journey and therapeutic outcomes in patients followed at a Brazilian public tertiary center — A retrospective cohort study
Source: PLoS One. 2026 Jul 28;21(7):e0353883. doi: 10.1371/journal.pone.0353883 (PMC13411926; doi:10.1371/journal.pone.0353883)
Supplement: S2 Table — Values indicate the number and proportion of patients within each therapeutic-response group who had previously received the listed immunosuppressive agent before the last clinical evaluation. (DOCX) [file pone.0353883.s002.docx]

**S2 Table. Prior immunosuppressive treatment by treatment-response group**

| **Prior treatment** | **Drug-responsive n = 113** | **Drug-refractory n = 21** | **Corticosteroid-dependent n = 16** |
| --- | --- | --- | --- |
| Azathioprine | 94 (83.2) | 18 (85.7) | 16 (100.0) |
| Methotrexate | 9 (8.0) | 13 (61.9) | 5 (31.2) |
| Cyclophosphamide | 0 | 4 (19) | 0 |
| Cyclosporine | 1 (0.9) | 10 (47.6) | 1 (6.2) |
| Mycophenolate | 3 (2.7) | 5 (23.8) | 0 |
| Rituximab | 0 | 4 (19) | 0 |
| Satralizumab | 1 (0.9) | 3 (14.3) | 1 (6.2) |
| Tocilizumab | 0 | 1 (4.8) | 0 |
| Prednisone | 100 (88.5) | 21 (100) | 16 (100) |

Values indicate the number and proportion of patients within each therapeutic-response group who had previously received the listed immunosuppressive agent before the last clinical evaluation. DR, drug-responsive; R, drug-refractory; C, corticosteroid-dependent.
