# Supplementary material for: Myasthenia gravis: Diagnostic journey and therapeutic outcomes in patients followed at a Brazilian public tertiary center — A retrospective cohort study
Source: PLoS One. 2026 Jul 28;21(7):e0353883. doi: 10.1371/journal.pone.0353883 (PMC13411926; doi:10.1371/journal.pone.0353883)
Supplement: S3 Table — Values represent the percentage of patients among those with at least one documented treatment-related adverse event (n = 67). More than one adverse event could be documented per patient. Events were grouped according to treatment exposure and known toxicity profiles as corticosteroid-associated adverse events, non-steroid immunosuppressant-associated adverse events, or infections during immunosuppressive therapy. These categories indicate clinical association in a retrospective chart review and do not imply definite causality. (DOCX) [file pone.0353883.s003.docx]

**S3 Table. Specific treatment-related adverse events**

| **Adverse event category** | **Specific adverse event** | **n/N (%)** |
| --- | --- | --- |
| Corticosteroid-associated adverse events | Hyperglycemia | 25/67 (37.3) |
|  | Osteoporosis/osteopenia | 12/67 (17.9) |
|  | Weight gain | 11/67 (16.4) |
|  | Dyslipidemia | 10/67 (14.9) |
|  | Cataract | 9/67 (13.4) |
|  | Glaucoma | 1/67 (1.5) |
|  | Central serous chorioretinopathy | 1/67 (1.5) |
| Non-steroid immunosuppressant-associated adverse events | Gastrointestinal symptoms | 9/67 (13.4) |
|  | Lymphopenia | 6/67 (9.0) |
|  | Allergic reaction | 3/67 (4.5) |
|  | Hepatotoxicity | 2/67 (3.0) |
|  | Basal cell carcinoma | 2/67 (3.0) |
|  | Renal dysfunction | 1/67 (1.5) |
| Infections during immunosuppressive therapy | Infection | 7/67 (10.4) |

Values represent the percentage of patients among those with at least one documented treatment-related adverse event (n = 67). More than one adverse event could be documented per patient. Events were grouped according to treatment exposure and known toxicity profiles as corticosteroid-associated adverse events, non-steroid immunosuppressant-associated adverse events, or infections during immunosuppressive therapy. These categories indicate clinical association in a retrospective chart review and do not imply definite causality.
