# Supplementary material for: Myasthenia gravis: Diagnostic journey and therapeutic outcomes in patients followed at a Brazilian public tertiary center — A retrospective cohort study
Source: PLoS One. 2026 Jul 28;21(7):e0353883. doi: 10.1371/journal.pone.0353883 (PMC13411926; doi:10.1371/journal.pone.0353883)
Supplement: S4 Table — Values represent the percentage of all reported misdiagnoses (n = 66). One patient reported more than one misdiagnosis. COPD, chronic obstructive pulmonary disease; GERD, gastroesophageal reflux disease. (DOCX) [file pone.0353883.s004.docx]

**S4 Table. Specific misdiagnoses in patients with myasthenia gravis**

| **Specific misdiagnosis** | **%** |
| --- | --- |
| Anxiety | 10.6 |
| Multiple sclerosis | 9.1 |
| Major depressive disorder | 7.6 |
| Guillain-Barré syndrome | 7.6 |
| Stroke | 7.6 |
| Functional Neurological Disorder | 7.6 |
| Peripheral facial palsy | 4.5 |
| Tendinopathy | 4.5 |
| Amyotrophic lateral sclerosis | 4.5 |
| Unspecified | 4.5 |
| Multiple cranial nerve syndrome | 3.0 |
| Brain neoplasm | 3.0 |
| Myopathy | 3.0 |
| Constitutional ptosis | 3.0 |
| Laryngeal neoplasia | 1.5 |
| Horner syndrome | 1.5 |
| Radiculopathy | 1.5 |
| Vertigo | 1.5 |
| Traumatic brain injury | 1.5 |
| Pneumonia | 1.5 |
| Glaucoma | 1.5 |
| Cerebral aneurysm | 1.5 |
| Cervical neoplasm | 1.5 |
| GERD | 1.5 |
| COPD | 1.5 |
| Arthropathy | 1.5 |
| Brainstem syndrome | 1.5 |
| Airway disorders | 1.5 |
| Anemia | 1.5 |

Values represent the percentage of all reported misdiagnoses (n = 66). One patient reported more than one misdiagnosis. COPD, chronic obstructive pulmonary disease; GERD, gastroesophageal reflux disease.
