# Supplementary material for: Myasthenia gravis: Diagnostic journey and therapeutic outcomes in patients followed at a Brazilian public tertiary center — A retrospective cohort study
Source: PLoS One. 2026 Jul 28;21(7):e0353883. doi: 10.1371/journal.pone.0353883 (PMC13411926; doi:10.1371/journal.pone.0353883)
Supplement: S5 Table — Values indicate the number of patients with available or classifiable information for each variable. Percentages represent data availability, not the frequency of the corresponding clinical finding. (DOCX) [file pone.0353883.s005.docx]

**S5 Table. Data availability and denominators for major variables, overall and by treatment group**

| **Variable** | **Overall available/classifiable, n/N (%)** | **DR available/classifiable, n/N (%)** | **C available/classifiable, n/N (%)** | **R available/classifiable, n/N (%)** | **Denominator used for percentage calculation** |
| --- | --- | --- | --- | --- | --- |
| Antibody status | 108/150 (72.0) | 75/113 (66.4) | 15/16 (93.8) | 18/21 (85.7) | Total cohort / total group; availability was defined as at least one documented antibody test result |
| Age at symptom onset | 150/150 (100.0) | 113/113 (100.0) | 16/16 (100.0) | 21/21 (100.0) | Total cohort / total group |
| Diagnostic delay | 131/150 (87.3) | 99/113 (87.6) | 13/16 (81.3) | 19/21 (90.5) | Patients with available questionnaire or chart data |
| Number of physicians consulted before diagnosis | 118/150 (78.7) | 88/113 (77.9) | 13/16 (81.3) | 17/21 (81.0) | Patients with available questionnaire or chart data |
| Previous misdiagnosis | 117/150 (78.0) | 88/113 (77.9) | 14/16 (87.5) | 15/21 (71.4) | Patients with available questionnaire or chart data |
| Myasthenic crisis or impending crisis at onset | 101/150 (67.3) | 80/113 (70.8) | 11/16 (68.8) | 10/21 (47.6) | Patients with available questionnaire or chart data |
| Hospitalization history | 113/150 (75.3) | 87/113 (77.0) | 11/16 (68.8) | 15/21 (71.4) | Patients with available questionnaire or chart data |
| Documented lifetime myasthenic crisis history/ impending crisis history | 139/150 (92.7) | 105/113 (92.9) | 14/16 (87.5) | 20/21 (95.2) | Patients with sufficiently informative longitudinal data |
| MGFA clinical class at last evaluation | 150/150 (100.0) | 113/113 (100.0) | 16/16 (100.0) | 21/21 (100.0) | Total cohort / total group |
| MGFA-PIS at last evaluation | 146/150 (97.3) | 110/113 (97.3) | 15/16 (93.8) | 21/21 (100.0) | Patients with sufficient follow-up data for MGFA-PIS classification |
| Documented clinically relevant adverse events | 141/150 (94.0) | 106/113 (93.8) | 15/16 (93.8) | 20/21 (95.2) | Patients with sufficiently informative longitudinal medical records |
| Race/color | 150/150 (100.0) | 113/113 (100.0) | 16/16 (100.0) | 21/21 (100.0) | Total cohort / total group |
| Sex | 150/150 (100.0) | 113/113 (100.0) | 16/16 (100.0) | 21/21 (100.0) | Total cohort / total group |

Values indicate the number of patients with available or classifiable information for each variable. Percentages represent data availability, not the frequency of the corresponding clinical finding. DR, drug-responsive; C, corticosteroid-dependent; R, drug-refractory; MGFA, Myasthenia Gravis Foundation of America; PIS, Postintervention Status.
