# Supplementary material for: Myasthenia gravis: Diagnostic journey and therapeutic outcomes in patients followed at a Brazilian public tertiary center — A retrospective cohort study
Source: PLoS One. 2026 Jul 28;21(7):e0353883. doi: 10.1371/journal.pone.0353883 (PMC13411926; doi:10.1371/journal.pone.0353883)
Supplement: S6 Table — Complete diagnostic-journey data were defined as availability of diagnostic delay, number of physicians consulted before diagnosis, and previous misdiagnosis. Complete cumulative outcome data were defined as availability of lifetime myasthenic crisis history, hospitalization history, and treatment-related adverse events. (DOCX) [file pone.0353883.s006.docx]

**S6 Table. Comparison of patients with complete and incomplete data for missingness pattern assessment**

| **Characteristic** | **Complete data** | **Incomplete data** | **p-value** |
| --- | --- | --- | --- |
| **Diagnostic journey data** | | | |
| Number of patients | 107 | 43 | — |
| Age at symptom onset, years, median (IQR) | 34.0 (26.5–49.5) | 36.0 (24.5–46.5) | 0.414 |
| Disease duration, years, median (IQR) | 9.8 (3.5–19.4) | 16.9 (10.6–22.5) | 0.002 |
| Female sex, n/N (%) | 67/107 (62.6) | 31/43 (72.1) | 0.344 |
| White race/color, n/N (%) | 80/107 (74.8) | 32/43 (74.4) | 1.000 |
| Generalized phenotype, n/N (%) | 98/107 (91.6) | 42/43 (97.7) | 0.282 |
| Documented AChR-positive MG, n/N (%) | 54/107 (50.5) | 20/43 (46.5) | 0.720 |
| Documented MuSK-positive MG, n/N (%) | 5/107 (4.7) | 3/43 (7.0) | 0.690 |
| Treatment group, overall distribution | — | — | 0.971 |
| DR, n/N (%) | 81/107 (75.7) | 32/43 (74.4) | — |
| C, n/N (%) | 11/107 (10.3) | 5/43 (11.6) | — |
| R, n/N (%) | 15/107 (14.0) | 6/43 (14.0) | — |
| **Cumulative outcome data** | | | |
| Number of patients | 110 | 40 | — |
| Age at symptom onset, years, median (IQR) | 37.0 (25.5–50.8) | 31.5 (24.5–44.5) | 0.182 |
| Disease duration, years, median (IQR) | 9.9 (3.7–19.3) | 15.4 (9.3–24.7) | 0.007 |
| Female sex, n/N (%) | 68/110 (61.8) | 30/40 (75.0) | 0.175 |
| White race/color, n/N (%) | 84/110 (76.4) | 28/40 (70.0) | 0.524 |
| Generalized phenotype, n/N (%) | 102/110 (92.7) | 38/40 (95.0) | 1.000 |
| Documented AChR-positive MG, n/N (%) | 56/110 (50.9) | 18/40 (45.0) | 0.582 |
| Documented MuSK-positive MG, n/N (%) | 5/110 (4.5) | 3/40 (7.5) | 0.440 |
| Treatment group, overall distribution | — | — | 0.652 |
| DR, n/N (%) | 85/110 (77.3) | 28/40 (70.0) | — |
| C, n/N (%) | 11/110 (10.0) | 5/40 (12.5) | — |
| R, n/N (%) | 14/110 (12.7) | 7/40 (17.5) | — |

Complete diagnostic journey data were defined as availability of diagnostic delay, number of physicians consulted before diagnosis, and previous misdiagnosis. Complete cumulative outcome data were defined as availability of lifetime myasthenic crisis history, hospitalization history, and clinically relevant adverse events. Continuous variables were compared using the Mann–Whitney U test and are presented as median (IQR). Categorical variables were compared using Fisher’s exact test, except for treatment-group distribution, which was compared using the chi-square test. The p-value for treatment group refers to the overall comparison of the DR/C/R distribution between patients with complete and incomplete data. DR, drug-responsive; C, corticosteroid-dependent; R, drug-refractory; AChR, acetylcholine receptor; MuSK, muscle-specific kinase; MG, myasthenia gravis.
