# Supplementary material for: Myasthenia gravis: Diagnostic journey and therapeutic outcomes in patients followed at a Brazilian public tertiary center — A retrospective cohort study
Source: PLoS One. 2026 Jul 28;21(7):e0353883. doi: 10.1371/journal.pone.0353883 (PMC13411926; doi:10.1371/journal.pone.0353883)
Supplement: S7 Table — The table details adequate corticosteroid exposure, adequately tested nonsteroidal immunosuppressive agents, clinical response, treatment-limiting adverse events or intolerance, and the final basis for refractory classification for each patient. (DOCX) [file pone.0353883.s007.docx]

**S7 Table. Individual criteria supporting classification of drug-refractory myasthenia gravis.**

| **Patient ID** | **Adequate corticosteroid trial** | **No. adequately tested IS agents** | **IS agents used** | **Clinical response after adequate therapy** | **Treatment-limiting AE or intolerance** | **Final basis for refractory classification** |
| --- | --- | --- | --- | --- | --- | --- |
| R001 | Yes | 2 | Cyclosporine; azathioprine | Partial improvement but persistent disabling symptoms | Yes | Both insufficient response and AE/intolerance |
| R002 | Yes | 4 | Azathioprine; cyclosporine; methotrexate; cyclophosphamide | Worsened | No | Insufficient clinical response despite adequate therapy |
| R003 | Yes | 3 | Azathioprine; methotrexate; cyclophosphamide | Worsened | No | Insufficient clinical response despite adequate therapy |
| R004 | Yes | 3 | Azathioprine; cyclosporine; methotrexate | Unchanged/no meaningful response | No | Insufficient clinical response despite adequate therapy |
| R005 | Yes | 2 | Cyclosporine; rituximab | Unchanged/no meaningful response | Yes | Both insufficient response and AE/intolerance |
| R006 | Yes | 3 | Cyclosporine; azathioprine; mycophenolate | Partial improvement but persistent disabling symptoms | Yes | Both insufficient response and AE/intolerance |
| R007 | Yes | 2 | Cyclosporine; azathioprine | Unchanged/no meaningful response | Yes | Both insufficient response and AE/intolerance |
| R008 | Yes | 3 | Rituximab; azathioprine; mycophenolate | Unchanged/no meaningful response | Yes | Both insufficient response and AE/intolerance |
| R009 | Yes | 4 | Rituximab; azathioprine; mycophenolate; methotrexate | Partial improvement but persistent disabling symptoms | Yes | Both insufficient response and AE/intolerance |
| R010 | Yes | 2 | Methotrexate; azathioprine | Partial improvement but persistent disabling symptoms | Yes | Both insufficient response and AE/intolerance |
| R011 | Yes | 2 | Azathioprine; mycophenolate | Partial improvement but persistent disabling symptoms | Yes | Both insufficient response and AE/intolerance |
| R012 | Yes | 2 | Cyclosporine; azathioprine | Recurrent exacerbations/rescue need | No | Insufficient clinical response despite adequate therapy |
| R013 | Yes | 5 | Azathioprine; cyclosporine; methotrexate; satralizumab; cyclophosphamide | Unchanged/no meaningful response | Yes | Both insufficient response and AE/intolerance |
| R014 | Yes | 2 | Azathioprine; mycophenolate | Partial improvement but persistent disabling symptoms | Yes | Both insufficient response and AE/intolerance |
| R015 | Yes | 5 | Azathioprine; cyclosporine; methotrexate; satralizumab; cyclophosphamide | Unchanged/no meaningful response | Yes | Both insufficient response and AE/intolerance |
| R016 | Yes | 2 | Azathioprine; cyclosporine | Partial improvement but persistent disabling symptoms | No | Insufficient clinical response despite adequate therapy |
| R017 | Yes | 3 | Azathioprine; methotrexate; rituximab | Unchanged/no meaningful response | Yes | Both insufficient response and AE/intolerance |
| R018 | Yes | 3 | Azathioprine; methotrexate; rituximab | Unchanged/no meaningful response | Yes | Both insufficient response and AE/intolerance |
| R019 | Yes | 2 | Azathioprine; cyclosporine | Partial improvement but persistent disabling symptoms | No | Insufficient clinical response despite adequate therapy |
| R020 | Yes | 3 | Azathioprine; cyclosporine; mycophenolate | Improved | No | Insufficient response after ≥2 IS; later improved with additional therapy |
| R021 | Yes | 2 | Azathioprine; mycophenolate | Partial improvement but persistent disabling symptoms | Yes | Both insufficient response and AE/intolerance |

All patients had an adequate corticosteroid trial and exposure to at least two nonsteroidal immunosuppressive agents at adequate dose and duration. Treatment-limiting adverse events or intolerance were considered only when they prevented continued use, dose optimization, or treatment escalation. AE, adverse event; IS, immunosuppressive agent.
