# Supplementary material for: Myasthenia gravis: Diagnostic journey and therapeutic outcomes in patients followed at a Brazilian public tertiary center — A retrospective cohort study
Source: PLoS One. 2026 Jul 28;21(7):e0353883. doi: 10.1371/journal.pone.0353883 (PMC13411926; doi:10.1371/journal.pone.0353883)
Supplement: S8 Table — Separate binary logistic regression models were fitted for each outcome and pairwise treatment-group comparison. Models included treatment-response group and disease duration in years. Adjusted odds ratios refer to the treatment-group comparison shown, with drug-responsive patients as the reference group. (DOCX) [file pone.0353883.s008.docx]

**S8 Table. Exploratory logistic regression analyses of cumulative therapeutic outcomes adjusted for disease duration.**

| **Outcome** | **Comparison** | **Events, n/N (%)** | **Adjusted OR (95% CI)** | **p-value** |
| --- | --- | --- | --- | --- |
| Hospitalization | R vs DR | R: 10/15 (66.7%); DR: 34/87 (39.1%) | 2.98 (0.93–9.54) | 0.066 |
| Hospitalization | C vs DR | C: 3/11 (27.3%); DR: 34/87 (39.1%) | 0.58 (0.14–2.37) | 0.452 |
| Documented myasthenic crisis | R vs DR | R: 9/20 (45.0%); DR: 22/105 (21.0%) | 3.32 (1.20–9.21) | 0.021 |
| Documented myasthenic crisis | C vs DR | C: 4/14 (28.6%); DR: 22/105 (21.0%) | 1.48 (0.42–5.18) | 0.541 |
| Impending myasthenic crisis | R vs DR | R: 5/20 (25.0%); DR: 28/105 (26.7%) | 0.96 (0.32–2.92) | 0.945 |
| Impending myasthenic crisis | C vs DR | C: 5/14 (35.7%); DR: 28/105 (26.7%) | 1.48 (0.45–4.81) | 0.516 |
| At least one treatment-related adverse event | R vs DR | R: 14/20 (70.0%); DR: 42/106 (39.6%) | 3.27 (1.14–9.31) | 0.027 |
| At least one treatment-related adverse event | C vs DR | C: 11/15 (73.3%); DR: 42/106 (39.6%) | 4.76 (1.39–16.28) | 0.013 |

Separate binary logistic regression models were fitted for each outcome and pairwise treatment-group comparison. Each model included treatment-response group and disease duration in years. Adjusted odds ratios refer to the first group listed in the comparison, with drug-responsive patients as the reference group. All p-values are two-sided.

Abbreviations: C, corticosteroid-dependent; CI, confidence interval; DR, drug-responsive; OR, odds ratio; R, drug-refractory.
